# Integrative multi-cohort analysis of DNA methylation profiles for pancreatic ductal adenocarcinoma biomarker discovery and prognosis

**DOI:** 10.3389/fbinf.2026.1808516

**Published:** 2026-06-03

**Authors:** Humera Inayat, Mohammad Aazam

**Affiliations:** Carnegie Mellon University in Qatar, Doha, Qatar

**Keywords:** biomarkers, diagnostic markers, epigenetic, machine learning, methylation, neurodevelopmental hubs, PDAC, perineural invasion

## Abstract

Pancreatic ductal adenocarcinoma (PDAC) remains one of the most lethal human malignancies, in part due to late diagnosis and the lack of robust molecular biomarkers. Although aberrant DNA methylation is a defining feature of PDAC, most studies rely on single cohorts, limiting reproducibility and biological interpretation. Here, we performed an integrative multi-cohort analysis of genome-wide DNA methylation profiles from four independent PDAC datasets generated on Illumina EPIC and HumanMethylation450 platforms, comprising 364 tumors and 99 normal controls. Using a harmonized preprocessing cross-platform normalization strategy, we identified hundreds of CpG sites that were consistently differentially methylated across all cohorts. Integration with pancreatic chromatin-state annotations, hydroxymethylation profiles, and protein–protein interaction networks was used to contextualize recurrent DNA methylation changes. This analysis showed that hypermethylation preferentially targets promoter- and enhancer-associated regulatory elements linked to neuronal and developmental gene networks. To assess predictive relevance, we trained interpretable and non-linear machine-learning models with strict cross-cohort evaluation, and combined SHAP-based feature attribution with deep neural network saliency analysis. Intersection of statistical, biological, and machine-learning evidence identified a compact 18-CpG candidate signature that stratified tumor and normal samples across the analyzed cohorts. Together, this study demonstrates that PDAC methylation remodeling exhibits consistent and reproducible patterns across cohorts that are biologically interpretable. Furthermore, the study shows that integrating chromatin context, network topology, and interpretable machine learning can help identify candidate epigenetic biomarkers with translational potential.

## 1 Introduction

According to World Health Organization data, pancreatic ductal adenocarcinoma (PDAC) remains a major global health burden, with the highest numbers of new cases and deaths reported in regions of Europe and the Americas (Siegel et al., 2025). Mortality from PDAC continues to rise, and in 2022 it ranked as the fourth leading cause of cancer-related death in Europe (Roškovičová et al., 2026). The combination of low incidence and non-specific early symptoms frequently results in late diagnosis, with many patients presenting with locally advanced or metastatic disease at the time of detection (Bugazia et al., 2024; Liu et al., 2024; Mukund et al., 2024). Most patients are diagnosed at advanced stages, partly because of compromised sensitivity and selectivity of the early-stage biomarkers (Bhattacharjee et al., 2025). Although CA19-9 is not sufficiently reliable for screening or primary diagnosis, its correlation with tumor burden supports its use in prognostic assessment and disease monitoring. When considered alongside CEA, these markers can aid in identifying advanced-stage PDAC and support multidisciplinary evaluation of tumor stage and resectability but not for the diagnosis of asymptomatic PDAC (Brancato et al., 2025; van Manen et al., 2020). In light of these diagnostic challenges, increasing attention has shifted toward molecular mechanisms underlying PDAC initiation and progression. Emerging evidence implicates epigenetic dysregulation, particularly aberrant DNA methylation, as a key driver of PDAC development, disease progression, and transcriptional reprogramming, offering a biologically grounded avenue for identifying more stable and informative biomarkers beyond conventional serum protein markers (Ding et al., 2025; Liu et al., 2024).

DNA methylation alterations represent a stable and heritable layer of the epigenome, making them particularly well suited for disease detection and longitudinal monitoring (Ding et al., 2025; Ehrlich, 2019). Aberrant promoter hypermethylation can lead to silencing of tumor-suppressor genes, while global or regional hypomethylation may activate oncogenic programs or destabilize chromatin architecture (Grafenhorst et al., 2025; Kan et al., 2019). Importantly, DNA methylation–based markers have already demonstrated clinical utility in other cancer settings, including disease monitoring, minimal residual disease detection, and assessment of treatment response using tissue and circulating cell-free DNA, underscoring their translational potential (Butler et al., 2020; Grawenda and O’Neill, 2015; Hariharan and Jenkins, 2020; Li et al., 2024; Xie et al., 2025; Zhao et al., 2024).

Despite the growing interest in DNA methylation as a biomarker in pancreatic ductal adenocarcinoma (PDAC), establishing reproducible methylation patterns across independent studies remains challenging (Grafenhorst et al., 2025; Liu et al., 2024). Although multiple datasets have been analyzed, many investigations rely on single cohorts or limited external validation, making it difficult to fully separate disease-associated methylation changes from platform-specific effects, cohort heterogeneity, or technical variability (Kasmirski et al., 2025).

To address this, we conducted a multi-cohort methylation analysis integrating four independent PDAC datasets generated using the Illumina EPIC and HM450K arrays. In addition to differential methylation analysis, we incorporated pancreatic chromatin-state annotations, cfDNA 5-hydroxymethylation data, protein–protein interaction networks, and interpretable machine-learning approaches. This integrative strategy was used to assess the consistency of methylation signals across datasets and to place recurrent CpG sites within their regulatory and biological context.

The aim of this study was to identify DNA methylation changes in PDAC that are consistently observed across independent cohorts and array platforms and to examine their functional and predictive relevance. By combining differential methylation analysis with chromatin-state information, hydroxymethylation profiling, network analysis, and cross-cohort machine-learning evaluation, we sought to prioritize CpG sites supported by multiple analytical approaches rather than to propose definitive diagnostic markers.

In this study, we demonstrate that a subset of PDAC-associated DNA methylation changes is consistently detectable across multiple independent cohorts and array platforms when analyzed within a unified, cross-cohort framework. Through integration of differential methylation analysis, pancreatic chromatin-state annotation, cfDNA 5-hydroxymethylation profiles, protein–protein interaction networks, and interpretable machine-learning models, we identified a compact panel of 18 CpG sites that is reproducibly altered across all four datasets and supported by both linear and non-linear predictive models. Rather than proposing an immediate diagnostic assay, this work shows that a small, interpretable CpG panel can be prioritized when statistical reproducibility, regulatory relevance, network context, and predictive contribution are considered jointly. Together, these findings indicate that PDAC-associated epigenetic alterations are structured rather than stochastic and that the identified 18-CpG set represents a stable and biologically grounded candidate marker panel for future functional and translational validation.

## 2 Materials and methods

### 2.1 Study cohorts and design

**FIGURE 1:**
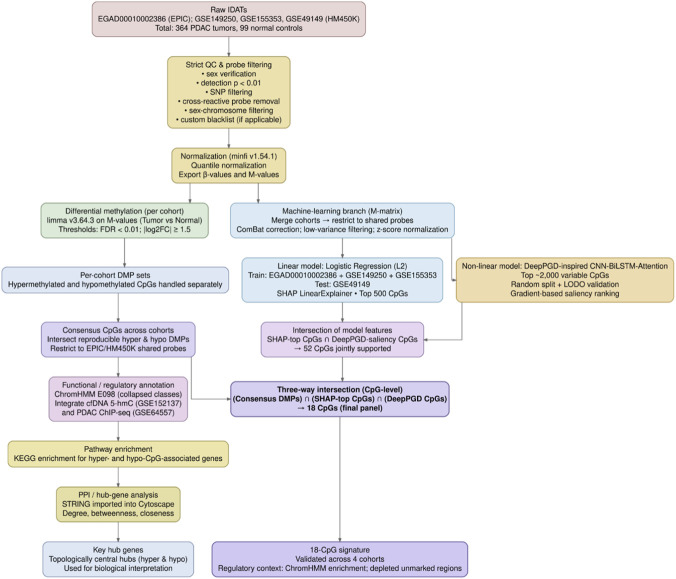
Overview of the integrative multi-cohort DNA methylation and machine-learning framework in PDAC. Raw IDAT files from four independent PDAC cohorts (EGAD00010002386, GSE149250, GSE155353, GSE49149) were processed using a harmonized pipeline including quality control, probe filtering, and normalization. Differential methylation analysis identified consensus hyper- and hypomethylated CpGs across cohorts, which were functionally annotated using pancreatic ChromHMM states and integrated with pathway and protein–protein interaction analyses. In parallel, batch-corrected M-values were analyzed using linear (logistic regression with SHAP) and non-linear (DeepPGD-inspired) machine-learning models. Intersection of differential methylation and model-prioritized features yielded a reproducible 18-CpG signature enriched in promoter- and enhancer-associated regulatory elements.

Four publicly available PDAC methylation datasets were analyzed: European Genome–phenome Archive (EGA), accession EGAD00010002386 https://ega-archive.org (Illumina EPIC) and GSE149250 (Gregório et al., 2020), GSE155353 (Endo et al., 2021), and GSE49149 (Bailey et al., 2016) (Illumina HM450K). Combined, these datasets include 364 PDAC tumors and 99 normal controls. A harmonized preprocessing workflow was used for cross-platform comparability (Figure 1).

### 2.2 Preprocessing of DNA methylation data

Raw IDAT files were processed in R using the *minfi* package (v1.54.1) (Aryee et al., 2014). Probes with detection p-values ≥0.01, probes containing single-nucleotide polymorphisms at CpG or extension sites, probes listed as cross-reactive (Zhou blacklist), and probes located on sex chromosomes were removed using the method described by Zhou et al. (2017). Quantile normalization produced β-values and M-values. For downstream machine-learning analyses, merged M-value matrices were batch-corrected using the ComBat method implemented in the *sva* package (v3.56.0) (Maksimovic et al., 2015). ComBat correction was applied after restricting analyses to probes shared across the Illumina EPIC and HM450K platforms to minimize platform- and cohort-specific technical variability while preserving biologically meaningful methylation differences.

### 2.3 Differential methylation and consensus CpG identification

Differential methylation analysis was performed using *limma* (v3.64.3) on M-values with thresholds of FDR <0.01 and |Δfc| ≥ 1.5 (Ritchie et al., 2015). Consensus CpGs were defined as those significant across all four datasets, restricted to probes shared by EPIC and HM450K.

### 2.4 Chromatin-state and 5-hydroxymethylation integration

Consensus CpGs were mapped to ChromHMM pancreatic tissue annotations (E098) and collapsed to promoter-like, enhancer-like, transcribed, repressed/heterochromatic, and quiescent categories (Li et al., 2016). PDAC ChIP-seq datasets (H3K27ac, H3K4me3, H3K9me3) were employed to define the regulatory context (Gulko and Siepel, 2019).

### 2.5 Protein–protein interaction network analysis

STRING database interactions were imported into Cytoscape using a confidence score threshold of ≥0.7 to generate protein–protein interaction networks for genes associated with consensus hypermethylated and hypomethylated CpGs. Network topology was analyzed in Cytoscape using the NetworkAnalyzer tool, and degree, betweenness, and closeness centrality metrics were computed to identify topologically important hub genes (Sanchez and Mackenzie, 2020).

### 2.6 Logistic regression with SHAP-Based feature attribution

To obtain an interpretable baseline classifier and quantify CpG-level contributions to PDAC status, a regularized logistic regression (LR) model was trained on harmonized DNA methylation data from three discovery cohorts (GSE149250, GSE155353, and EGAD00010002386). Preprocessing included probe-level quality control, removal of probes located on sex chromosomes and known cross-reactive regions, batch correction across platforms, and filtering of low-variance CpGs (variance <0.01) (Aryee et al., 2014; Ritchie et al., 2015; Zhou et al., 2017). The GSE49149 cohort was entirely withheld from model training and used exclusively as an independent evaluation set to assess cross-cohort generalization. The LR model was implemented with L2 regularization and class-balanced weights to account for group imbalance between PDAC and normal samples.

Logistic regression was selected as a transparent baseline model to enable direct biological interpretation of CpG-level effects, while maintaining robustness in high-dimensional settings (Ponce-Bobadilla et al., 2024). Feature attribution was performed using SHAP LinearExplainer, which provides additive explanations consistent with the fitted regression coefficients and remains stable in the presence of correlated predictors commonly observed in DNA methylation data. SHAP values were computed for each CpG following the method described by Ponce-Bobadilla on a per-sample basis and summarized as mean absolute SHAP values across the training set to rank features according to their contribution to PDAC versus normal classification (Ponce-Bobadilla et al., 2024).

The top 500 CpGs with the largest mean absolute SHAP values were retained as the most influential features. These probes were annotated using curated Illumina HM450K and EPIC manifest files, and gene-level SHAP scores were derived by averaging SHAP values across all CpGs mapping to the same gene. Although SHAP values reflect feature contributions within the trained model rather than causal effects, this framework provides a principled and reproducible approach for identifying CpG- and gene-level biomarkers with consistent predictive relevance across cohorts.

Model performance was evaluated on the held-out GSE49149 dataset using receiver operating characteristic area under the curve (ROC AUC), precision–recall AUC (PR-AUC), accuracy, and Matthews correlation coefficient (MCC), providing a stringent assessment of generalization across independent methylation cohorts.

### 2.7 DeepPGD-inspired neural network and non-linear feature ranking

To complement the linear LR–SHAP framework and capture potential non-linear dependencies among CpG sites, a deep neural network inspired by the DeepPGD (Teragawa et al., 2024) architecture was implemented. The network comprised a one-dimensional convolutional layer (64 filters), a bidirectional LSTM module (64 units), an attention mechanism and fully connected layers with dropout regularization. Within the merged discovery cohorts, CpGs were ranked by variance and the top 2,000 most variable probes were selected as inputs. The model was trained for 40 epochs using the Adam optimizer (batch size 32) with binary cross-entropy loss (Teragawa et al., 2024).

Performance was evaluated under two regimes: (i) an 80/20 random split within the merged discovery data and (ii) a leave-one-dataset-out setting in which EGAD00010002386 served as an external test cohort. Gradient-based saliency maps were used to obtain attribution scores for each input CpG. As for the LR–SHAP pipeline, CpG-level scores were aggregated to gene level by averaging attributions across probes mapping to the same gene. The random-split configuration yielded clearer separation between tumor and normal samples and more stable attribution patterns across training runs; therefore, gene-level features derived from this setting were used for integration with SHAP-prioritised genes and differentially methylated loci. We used random-split attribution for stability of saliency patterns and retained cross-cohort testing to assess generalization.

### 2.8 Integration of machine-learning features with differential methylation

**FIGURE 2:**
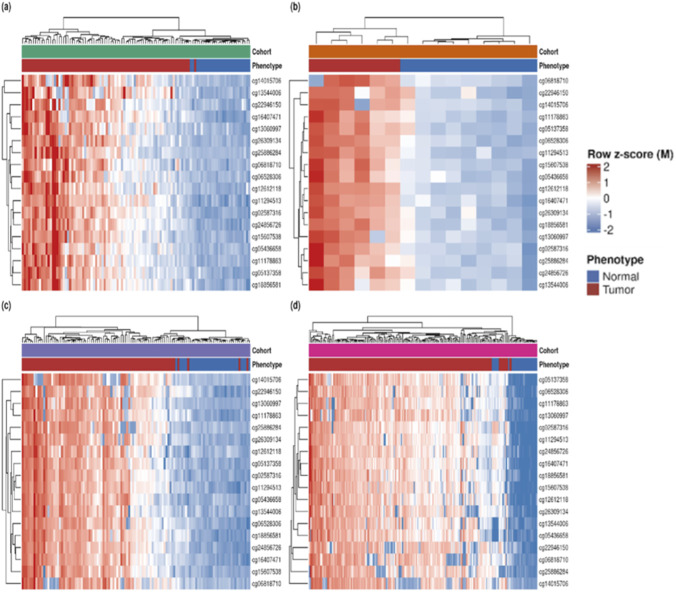
Multi-cohort PDAC DNA methylation signature defined by 18 CpG sites. Heatmaps show the row-wise z-scored ComBat-corrected methylation M-values for the 18-CpG panel across four independent cohorts: **(a)** EGAD00010002386, **(b)** GSE149250, **(c)** GSE155353, and **(d)** GSE49149. Columns represent individual samples and rows represent CpG sites; hierarchical clustering was applied within each cohort. The top annotation bars indicate cohort and phenotype (Normal vs. Tumor). Color scale denotes relative methylation (red = higher, blue = lower) after per-CpG standardization, illustrating cohort-consistent patterns of tumor–normal separation. This visualization is intended for descriptive purposes and does not constitute a quantitative assessment of classification robustness, which is instead evaluated using held-out and external-cohort model performance.

Gene sets derived from the two machine-learning frameworks were next integrated with the cross-cohort differential methylation results. The SHAP-based LR model yielded the top 500 CpGs ranked by mean absolute SHAP values, and the DeepPGD-like model highlighted approximately 2000 CpGs based on saliency-derived attributions. Intersecting these lists produced 52 CpGs jointly supported by linear and non-linear models. To refine this integration at higher resolution, we performed a three-way intersection at the CpG level, combining CpGs prioritized by SHAP (linear model), DeepPGD (non-linear model), and consensus differential methylation (logFC ±1.5, FDR ≤0.01). This yielded 18 core CpG sites with consistent signals across all methods, forming the basis of the final heatmap signature (Figure 2). These CpGs stratify tumor from normal samples across four cohorts and represent a stability-focused set of candidate CpGs supported by both statistical and machine-learning analyses.

We mapped these 18 CpGs to their associated genes using curated HM450K and EPIC probe annotations. The resulting gene list comprised: ZNF382, LRRC38, IRF4, ZSCAN23, NPBWR1, C9orf50, HSD17B12, ASCL4, CHFR, CCNA1, MDGA2, SH3GL3, PRKCB, ZFP82, AC006116.21, AC003006.7, FER1L4, ZNF529, RP4-597A16.2, NTMT1, MIR129-2, ZNF542P, ZNF154, ZSCAN5A, ZNF551. This gene set represents CpGs consistently prioritized across both machine-learning and statistical frameworks, grounded in both differential methylation and model-based interpretability. While this intersection-based strategy identifies CpGs that are consistently observed across cohorts, it may preferentially select highly stable features and does not necessarily capture the most biologically impactful or cohort-specific methylation events. Additionally, since the analysis was performed on harmonized public datasets, residual cohort-specific effects and shared preprocessing structure cannot be fully excluded.

## 3 Results

Our analysis revealed distinct DNA methylation profiles in PDAC, characterized by widespread hypermethylation at CpG islands and hypomethylation in intergenic regions. Our analysis of genome-wide methylation profiles in pancreatic ductal adenocarcinoma leveraged data from four independent cohorts (EGAD00010002386, GSE149250, GSE155353, and GSE49149) after rigorous quality control and probe filtering. The characteristics of these cohorts are summarized in Table 1.

**TABLE 1:** Summarizes the characteristics of the datasets employed in this study.

Dataset ID	Platform	Sample type	Cases (n)	Controls (n)
EGAD00010002386	EPIC	Tumor tissue/adjacent normal tissue	79	27
GSE149250	HM450K	Tumor tissue/adjacent normal tissue	6	9
GSE155353	HM450K	Tumor tissue/adjacent normal tissue	82	34
GSE49149	HM450K	Tumor tissue/adjacent normal tissue	167	29

### 3.1 Cross-cohort differential methylation and functional context

**FIGURE 3:**
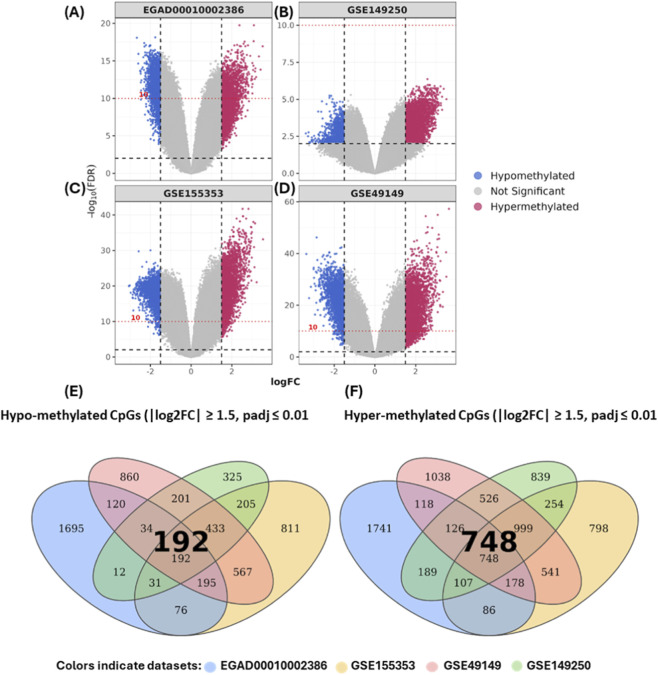
Volcano plots showing ΔM (difference in M-values) versus −log_10_ FDR for each dataset **(A–D)**. Hypermethylated CpGs (log2fc > 1.5) are shown in red, hypomethylated CpGs (log2fc < −1.5) in blue. Dashed vertical lines mark ±1.5 ΔM and the horizontal line indicates FDR = 0.01. **(E)** Four-set Venn diagram of hypomethylated CpGs; **(F)** Venn diagram of hypermethylated CpGs. Distinct colors correspond to each dataset. Numerical overlaps are displayed in each region, and the all-four intersection is bolded.

Across individual datasets, a substantial number of CpGs were identified as significantly differentially methylated based on criteria of an FDR <0.01 and an absolute log_2_ fold-change ≥1.5. Both hyper- and hypomethylated probes were observed across datasets, as illustrated by Volcano plot indicating widespread epigenetic alterations potentially associated with PDAC (Figures 3A–D). Across all datasets, extensive differential methylation was observed. Intersection identified 748 hypermethylated and 192 hypomethylated probes (Figures 3E,F). To further contextualize these shared methylation patterns within a regulatory and functional framework, we examined their regulatory distribution and pathway associations.

**FIGURE 4:**
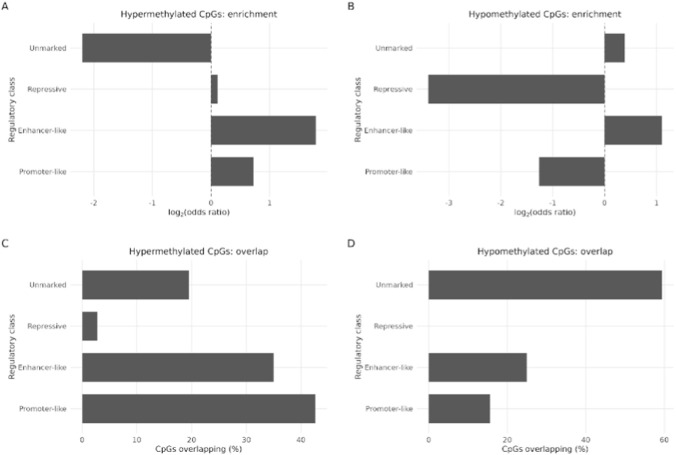
ChromHMM regulatory-class enrichment of differentially methylated CpGs across PDAC cohorts. ChromHMM-based enrichment analysis of CpGs consistently differentially methylated across four independent PDAC cohorts using the pancreatic ChromHMM E098 model. **(A)** Log_2_ odds ratios for enrichment of hypermethylated CpGs across promoter-like, enhancer-like, repressive, and unmarked chromatin states. **(B)** Log_2_ odds ratios for enrichment of hypomethylated CpGs across the same regulatory classes. **(C)** Percentage overlap of hypermethylated CpGs within each regulatory class. **(D)** Percentage overlap of hypomethylated CpGs within each regulatory class. The dashed vertical line indicates no enrichment (log_2_ odds ratio = 0). Enrichment was calculated relative to the background distribution of all HM450 CpGs.

Chromatin state enrichment analysis showed that hypermethylated CpGs were preferentially localized within promoter-like and repressive regions (Figure 4A), showing positive log_2_ (odds ratio) values for these classes. In contrast, enhancer-like and unmarked regions were underrepresented among hypermethylated sites. The corresponding overlap summary (Figure 4C) was consistent with this pattern, with more than 60% of hypermethylated CpGs mapping to repressive chromatin, followed by smaller proportions in unmarked and promoter-associated regions.

Conversely, hypomethylated CpGs exhibited a contrasting pattern (Figure 4B), with enrichment in promoter-like chromatin states (positive log_2_ (odds ratio) ≈ 2) and depletion in repressive and unmarked regions (negative log_2_ (odds ratio)). The overlap summary (Figure 4D) supported this observation, showing that nearly 70% of hypomethylated CpGs fell within promoter-associated regions, whereas enhancer- and repressive-like regions contributed minimally.

**FIGURE 5:**
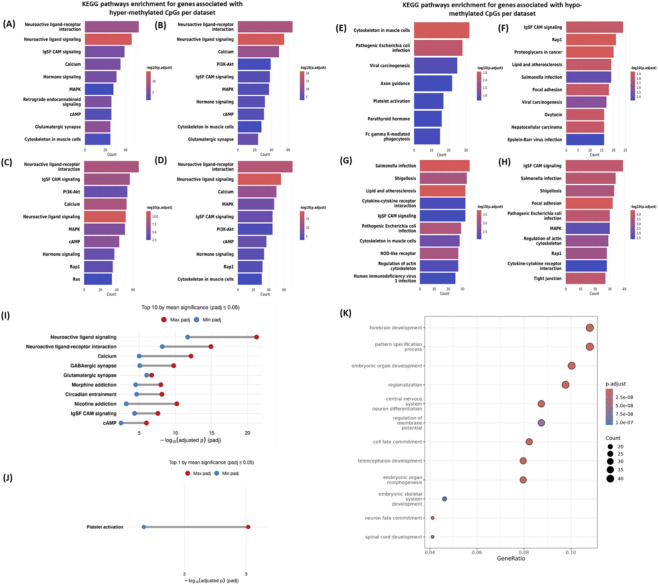
KEGG pathway enrichment analysis of genes associated with consensus differentially methylated CpGs across four PDAC cohorts. **(A–D)** Enriched pathways for genes associated with hypermethylated CpGs in EGAD00010002386, GSE149250, GSE155353, and GSE49149. **(E–H)** Corresponding KEGG enrichment for hypomethylated CpG-associated genes. Bar length represents gene count per pathway, and color indicates statistical significance (−log10 adjusted p-value). **(I,J)** Cross-cohort summary of reproducible pathways, showing the range of −log10 (adjusted p-values) across datasets. Only pathways with adjusted p-value <0.05 were considered significant. Comparison with cohort-specific enrichment analyses (Figure 6) shows that similar biological pathways are observed across datasets, although with greater variability, supporting the interpretation that consensus CpGs represent a reproducible core subset of PDAC-associated epigenetic alterations. **(K)** GO Biological Process enrichment for genes associated with hypermethylated CpGs shared across four cohorts. Dot size represents gene count, color indicates adjusted p-value, and the x-axis shows GeneRatio. It is important to note that no significant GO Biological Process terms were identified for hypomethylated CpG-associated genes under the significance threshold (FDR <0.05).

To further characterize the potential biological context of DNA methylation changes identified across cohorts, KEGG pathway enrichment analysis was performed separately for genes associated with consensus hypermethylated and consensus hypomethylated CpG sites. Across the four PDAC cohorts, genes associated with hypermethylated CpGs were enriched for multiple signaling pathways implicated in pancreatic cancer biology, including PI3K–Akt, MAPK, Rap1, Wnt signaling, ECM–receptor interaction, calcium signaling, and neuroactive ligand–receptor interaction (Figures 5A–D). To further characterize the biological processes associated with recurrent methylation changes, Gene Ontology (GO) enrichment analysis was performed using the genes associated with significantly hyper and hypomethylated CpGs across all datasets. Genes associated with hypermethylated CpGs showed significant enrichment for developmental and neurogenic processes, including forebrain development, pattern specification, embryonic organ development, regionalization, and central nervous system neuron differentiation (Figure 5K). Additional terms related to membrane potential regulation and cell fate commitment are consistent with enrichment of lineage-associated developmental and regulatory programs. In contrast, no GO Biological Process terms reached statistical significance for genes associated with hypomethylated CpGs under the selected threshold (FDR <0.05), consistent with the more heterogeneous distribution of hypomethylation observed across chromatin states and pathways. To assess cross-cohort consistency, pathway-level enrichment results were summarized across datasets and visualized using a node-based representation (Figure 5I). The resulting network shows enrichment across multiple pathways, consistent with association to interconnected signaling and regulatory processes in PDAC. Genes associated with hypomethylated CpGs showed a different enrichment pattern. Although several pathways reached significance in individual cohorts (Figures 5E–H), cross-cohort aggregation identified platelet activation as the only pathway consistently enriched across all datasets for hypomethylated CpGs associated genes (Figure 5J). Accordingly, the node-based summary for hypomethylated CpGs comprises a single pathway, reflecting more limited reproducibility of hypomethylation-associated pathway signals across cohorts.

**FIGURE 6:**
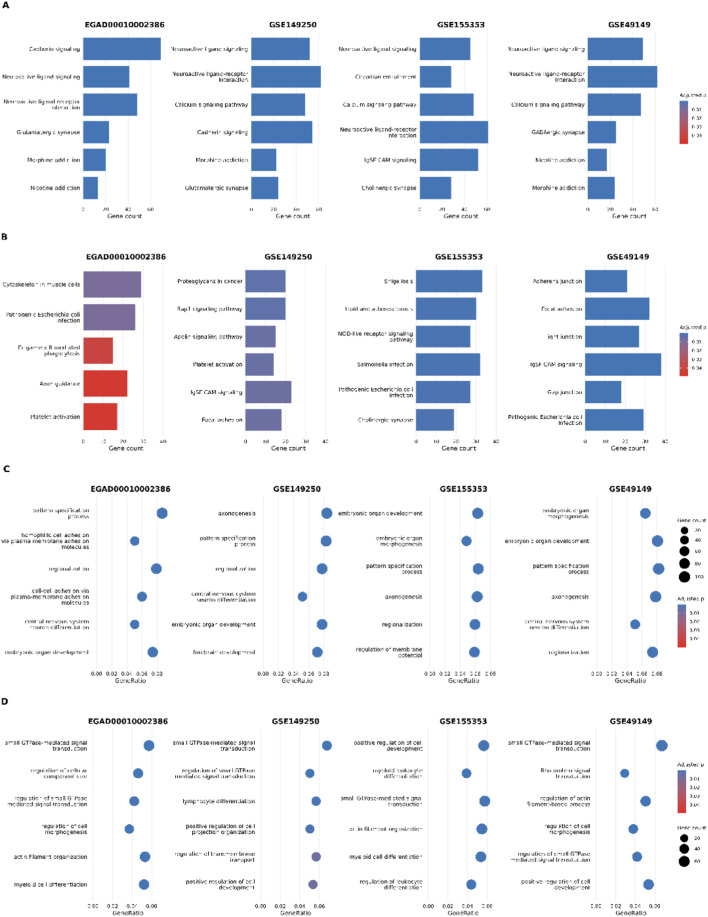
Comparative enrichment analysis of genes associated with cohort-specific differentially methylated CpGs across four PDAC cohorts. **(A)** KEGG enrichment of genes associated with cohort-specific hypermethylated CpGs in EGAD00010002386, GSE149250, GSE155353, and GSE49149. **(B)** KEGG enrichment of genes associated with cohort-specific hypomethylated CpGs across the same datasets. **(C)** GO Biological Process enrichment of genes associated with cohort-specific hypermethylated CpGs. **(D)** GO Biological Process enrichment of genes associated with cohort-specific hypomethylated CpGs. Within each subpanel, individual plots correspond to one dataset. In KEGG plots, bar length represents gene count and fill color indicates adjusted p-value. In GO plots, dot size represents gene count and dot color indicates adjusted p-value.

We have also evaluated whether the intersection-based strategy preferentially captures highly stable but not necessarily biologically meaningful CpGs, by performing pathway enrichment analysis on genes associated with cohort-specific differentially methylated CpGs in each dataset (Figure 6). Across all four cohorts, genes associated with cohort-specific hypermethylated CpGs showed enrichment for biological processes and pathways similar to those identified for consensus CpGs and for all differentially hyper and hypomethylated CpGs including the ones shared among the four (Figure 6), including neurodevelopmental processes, neuronal differentiation, axonogenesis, and signaling pathways such as calcium signaling, MAPK signaling, and neuroactive ligand–receptor interaction. These enrichment patterns were consistently observed across datasets despite differences in cohort composition and platform. However, compared with consensus CpGs, cohort-specific CpGs exhibited greater variability in enrichment profiles, particularly for hypomethylated loci, which showed heterogeneous pathway associations related to cytoskeletal organization, immune signaling, and stress-response pathways.

### 3.2 Network and regulatory context of key methylation-associated hub genes

Network centrality analysis revealed NRXN1 as the dominant hypermethylated hub, ranking highest in degree (88), betweenness (0.134), and closeness (0.40). Other high-degree hubs included GRIA2, SOX1, SLC32A1, OLIG2, and FOXG1. Betweenness identified bottleneck regulators such as NCAM1, CDH2, HOXB4, ZIC1, and NEUROD1. Closeness centrality highlighted globally influential hubs including RBFOX3, TBR1, NKX2-1, GRIA4, and SOX1. Altogether, the convergence of degree, betweenness, and closeness metrics identified a core set of neuronal and developmental regulators such as NRXN1, GRIA2, SOX1, OLIG2, FOXG1, NCAM1, and CDH2, as the key hypermethylation-driven hubs in PDAC.

**FIGURE 7:**
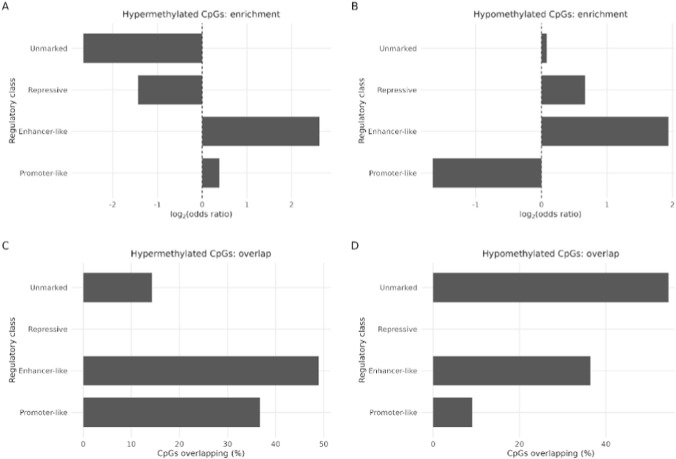
ChromHMM regulatory-class enrichment of key hub CpGs identified by integrative machine-learning and differential methylation analyses. ChromHMM regulatory annotation of key hub CpGs jointly supported by machine-learning feature selection and cross-cohort differential methylation analysis. Top panels depict log_2_ odds ratio enrichment of hypermethylated **(A)** and hypomethylated **(B)** hub CpGs across promoter-like, enhancer-like, repressive, and unmarked chromatin states from the pancreatic ChromHMM E098 model, with the dashed line indicating no enrichment. Bottom panels show the proportion of CpGs overlapping each regulatory class for hypermethylated **(C)** and hypomethylated **(D)** hub CpGs. Compared with the full CpG set, key hub CpGs demonstrate a more pronounced regulatory bias, reflecting their functional relevance and prioritization by both statistical and machine-learning frameworks.

ChromHMM-based annotation of CpGs associated with hypomethylated key hub genes revealed a structured but heterogeneous regulatory landscape. Several CpGs linked to metabolic and signaling-related genes, including MCCC2, DUSP1, TNFRSF10A, and CPT1A, were located within enhancer-like chromatin states (6_EnhG and 7_Enh), often mapping to TSS-proximal or gene body regions (Figure 7).

In contrast, CpGs associated with transcriptional regulators and stress-response genes such as GMPR, DGKI, MOGAT2, PML, and MAZ were primarily annotated to quiescent or weak transcriptional states. Only RUNX1 displayed hypomethylation at an active promoter state (1_TssA), consistent with a more direct promoter-level regulatory mechanism. Collectively, these observations indicate that hypomethylation of key hub genes in PDAC occurs across multiple chromatin contexts, encompassing enhancer-like regions, promoter-flanking domains, and quiescent chromatin.

ChromHMM annotation of CpGs associated with hypermethylated key hub genes revealed a strong enrichment within enhancer-like and promoter-flanking chromatin states, with comparatively fewer CpGs mapping to quiescent regions (Figure 7). A substantial fraction of hypermethylated CpGs localized to enhancer-associated states (7_Enh) and were linked to genes involved in neuronal differentiation and cell identity, including CDH2, DRD2, EOMES, NEUROD1, NKX2-1, OLIG2, and SOX1. In parallel, multiple CpGs associated with synaptic and signaling genes, most notably GRIA4, GRM1, NCAM1, NPY, and TBR1 were mapped to TSS-flanking promoter states (2_TssAFlnk), frequently within TSS200-proximal regions. A smaller subset of CpGs, including those linked to FOXG1 and NRXN1, were annotated to weakly transcribed or quiescent chromatin states. For direct comparison, representative hypermethylated and hypomethylated hub genes highlighted by the network analysis are summarized in Table 2.

**TABLE 2:** Representative hub-gene classes highlighted by the PDAC network analysis.

Methylation class	Representative hub genes	Dominant biological/regulatory context
Hypermethylated	NRXN1, GRIA2/GRIA4, SOX1, OLIG2, FOXG1, NCAM1, CDH2, GDNF, NEUROD1, TBR1	Neurodevelopmental, synaptic, and adhesion-related hubs enriched in promoter-like and enhancer-like regulatory elements
Hypomethylated	CPT1A, MCCC2, DUSP1, TNFRSF10A, PML, MAZ, RUNX1	Metabolic, stress-response, apoptotic, and transcriptional hubs distributed across mixed enhancer-like, promoter, and quiescent contexts

### 3.3 Machine-learning prioritization and regulatory characterization of the 18-CpG signature

To identify a stable and interpretable DNA methylation signature associated with PDAC, we integrated CpG-level evidence from three complementary approaches: (i) consensus differential methylation analysis using limma, retaining CpG sites with |log_2_FC| ≥ 1.5 and FDR ≤0.01 across all four PDAC cohorts (GSE149250, GSE155353, EGAD00010002386, and GSE49149); (ii) SHAP-attributed features from a logistic regression classifier trained on the discovery cohorts and evaluated on GSE49149; and (iii) gradient-based saliency scores derived from a DeepPGD-inspired convolutional–LSTM neural network trained on high-variance CpGs.

For the deep learning model, samples were randomly split into 80% training and 20% testing sets under an in-distribution evaluation setting, with EGAD samples included in both subsets. Model performance was assessed using standard classification metrics, yielding ROC AUC values between 0.88 and 0.90, a precision–recall AUC of 0.96, overall accuracy of 0.87, and a Matthews correlation coefficient of 0.64. Predicted probabilities for tumor samples were generally higher than those for normal samples, and training curves indicated stable optimization without marked divergence between training and testing loss.

Intersecting CpGs prioritized by all three approaches resulted in a set of 18 CpG probes consistently supported by statistical differential methylation, linear-model explainability, and neural network saliency attribution. These CpGs were retained as a high-confidence feature set for downstream characterization.

Visualization of this 18-CpG set using Z-score–normalized methylation values showed apparent separation between tumor and normal samples across all four cohorts (Figure 2). The observed pattern was dominated by hypermethylation in tumor samples, with a smaller subset of CpGs showing consistent hypomethylation. These trends were preserved across cohorts despite differences in platform and sample composition. However, this clustering is presented as a descriptive visualization and should not be interpreted as an independent measure of classification robustness. While the intersection of CpGs across statistical and machine-learning approaches enhances cross-cohort reproducibility, it may preferentially select features that are consistently detectable rather than those that capture context-specific or subtype-specific biological variation. Hence, the selected CpGs are intended to represent a potentially reproducible core signature rather than an exhaustive set of all biologically relevant methylation changes.

**FIGURE 8:**
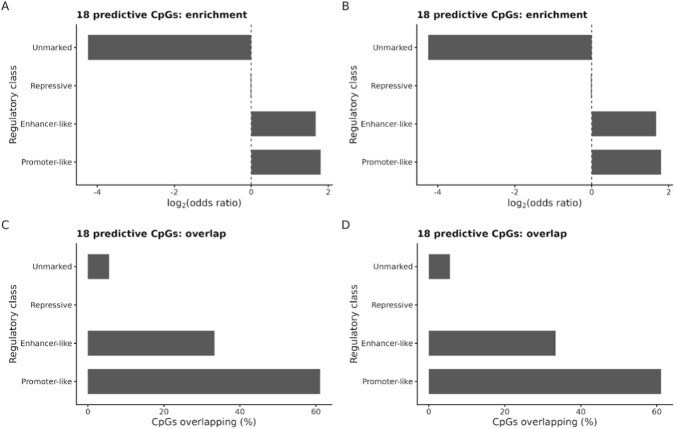
ChromHMM regulatory-class enrichment of the 18-CpG predictive signature. **(A,B)** Log_2_ odds-ratio enrichment of the 18 CpGs across promoter-like, enhancer-like, repressive, and unmarked chromatin states relative to the genomic background. The dashed line indicates no enrichment. **(C,D)** Proportion of CpGs overlapping each regulatory class. The predictive CpGs are significantly enriched in promoter- and enhancer-associated chromatin and depleted from unmarked regions, indicating preferential localization to transcriptionally relevant regulatory elements.

To evaluate whether the machine-learning–selected CpGs preferentially localize to specific regulatory contexts, we examined their distribution across pancreatic ChromHMM regulatory classes. The 18 CpGs were enriched in promoter-like and enhancer-associated regions, with minimal representation in unmarked or repressive chromatin states (Figure 8). Overlap analysis indicated that most CpGs in the signature mapped to promoter-associated regions, with a substantial fraction located within enhancer-like elements.

Taken together, CpGs prioritized by the combined machine-learning and statistical framework are interestingly found not to be randomly distributed across the genome but are enriched in regulatory elements with established roles in gene expression control. This supports the interpretability of the selected features and is consistent with their involvement in PDAC-associated epigenetic alterations.

## 4 Discussion

In this work, we performed a multi-cohort analysis of DNA methylation alterations in pancreatic ductal adenocarcinoma (PDAC) using four independent datasets generated on EPIC and HM450K platforms. By integrating differential methylation analysis with chromatin-state annotation, protein–protein interaction (PPI) network analysis, and interpretable machine-learning approaches, we aimed to characterize recurrent epigenetic changes in PDAC and to identify CpG sites that are both biologically meaningful and predictive.

### 4.1 Recurrent methylation changes define a structured epigenomic landscape in PDAC

Across all four cohorts, we identified 748 hypermethylated and 192 hypomethylated CpGs that were consistently differentially methylated in PDAC relative to normal tissue. The reproducibility of these CpGs across platforms and cohorts indicates a high degree of statistical consistency, suggesting that they represent stable disease-associated methylation changes rather than dataset-specific effects.

Annotation of these CpGs using ChromHMM states revealed a clear difference between hypermethylated and hypomethylated loci. Hypermethylated CpGs were enriched in promoter-associated and Polycomb-repressed regions, whereas hypomethylated CpGs were more frequently located in promoter-like chromatin and regions associated with 5-hydroxymethylation. This pattern is consistent with established models of epigenetic regulation, in which promoter-associated hypermethylation is linked to transcriptional repression and hypomethylation may be associated with gene activation or regulatory plasticity. However, the present analysis does not directly assess transcriptional consequences at individual loci and should therefore be interpreted as indicative of potential regulatory context rather than definitive functional effects. Pathway enrichment analysis further supported this organization, with recurrent methylation changes affecting PI3K–Akt, MAPK, Rap1, ECM–receptor interaction, and Wnt signaling pathways. These pathways are well established in PDAC biology and reflect processes such as growth signaling, cell adhesion, and interaction with the tumor microenvironment (Faleiro et al., 2021; He et al., 2019; Stanciu et al., 2022). The GO enrichment analysis further supports the structured nature of hypermethylation observed in PDAC. The enrichment of developmental and neurogenic biological processes aligns with the chromatin-state and network analyses, which collectively indicate preferential targeting of lineage-specific regulatory programs. The convergence of GO, KEGG, and ChromHMM analyses on neuronal and developmental processes suggests that PDAC-associated hypermethylation reflects coordinated repression of differentiation-related transcriptional networks.

In contrast, the absence of significant GO enrichment for hypomethylated CpG-associated genes reinforces their heterogeneous regulatory distribution and reduced cross-cohort consistency, suggesting that hypomethylation may reflect more context-dependent or dispersed regulatory changes rather than coherent biological programs.

Together, these results indicate that PDAC-associated methylation changes are proposing a potential underlying regulatory architecture. However, it is important to distinguish statistical and regulatory association from functional causality. The observed differential methylation and pathway enrichment patterns do not directly demonstrate that these CpG changes alter gene expression or cellular phenotypes and should therefore be interpreted as identifying candidate regulatory loci rather than functionally validated drivers of PDAC biology.

These findings can be further contextualized within emerging multi-omics frameworks of cancer biology, in which genomic, epigenomic, transcriptomic, and network-level features collectively contribute to oncogenic regulation. In this context, the present study captures the epigenetic layer of this hierarchy, integrating DNA methylation with chromatin-state annotation and network topology, while recognizing that establishing direct oncogenic relevance will require integration with additional molecular data types.

### 4.2 Network-central hub genes highlight epigenetic repression of neurodevelopmental programs

**FIGURE 9:**
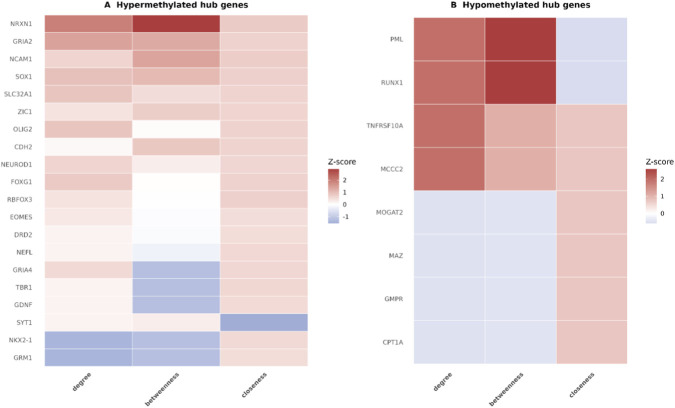
Network centrality analysis of hypermethylated and hypomethylated hub genes in PDAC. **(A)** Hypermethylated hub genes identified from the protein–protein interaction network and ranked based on combined Z-scored centrality measures (degree, betweenness, and closeness). The top 20 genes were selected using a stringent cutoff based on aggregated centrality (hub score), reflecting strong and consistent network connectivity. **(B)** Hypomethylated hub genes identified using a relaxed selection strategy due to lower overall network connectivity and reduced variance in centrality measures. The top 6–8 genes were selected based on hub_score ranking, without applying strict Z-score thresholds. Heatmaps display Z-score–normalized centrality values for degree, betweenness, and closeness. Red indicates higher relative centrality, while blue indicates lower centrality within each group.

To assess the potential functional importance of these methylation changes, we examined the network topology of genes associated with recurrently differentially methylated CpGs. PPI network analysis showed that methylation changes were concentrated in a limited number of highly connected genes rather than being evenly distributed across the network (Figure 9).

**FIGURE 10:**
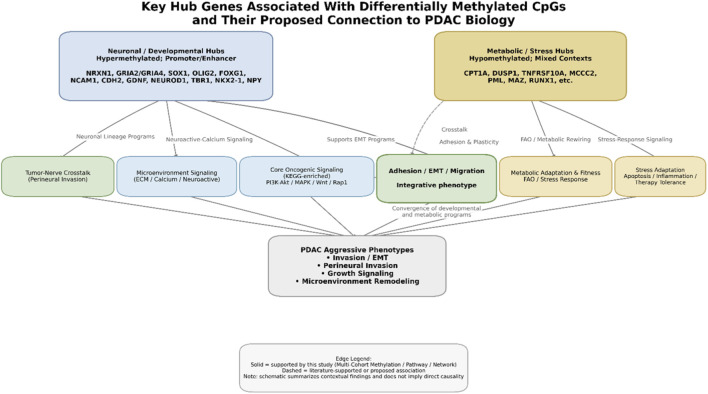
Schematic overview of key hub-gene modules associated with differentially methylated CpGs in PDAC. Neurodevelopmental and metabolic/stress hub genes identified from multi-cohort DNA methylation analysis are shown with their proposed functional connections to oncogenic signaling, adhesion/EMT, metabolic adaptation, and stress-response pathways, converging on aggressive PDAC phenotypes. Solid edges denote associations supported by this study, while dashed edges indicate literature-supported or proposed links.

A striking feature of the hypermethylated hubs was their enrichment for genes involved in neurodevelopment, neuronal signaling, and cell adhesion. These included transcription factors (ZIC1, ZIC4, SOX family members, OLIG2, NKX2-1), neurotransmission-related genes (GRIA2, GRIA4, NPY), and adhesion or neurotrophic factors (CDH2, NCAM1, GDNF). Many of these genes showed promoter- or enhancer-associated hypermethylation across multiple CpGs, suggesting coordinated epigenetic repression (Figure 10).

This pattern is notable given the prominent role of neural interactions in PDAC, particularly perineural invasion (Felsenstein et al., 2022). These observations may also have implications for tumor–microenvironment interactions, including immune modulation, as neural and adhesion-related signaling pathways have been linked to immune evasion and T-cell activity in solid tumors.

The observed hypermethylation may reflect suppression of alternative lineage or differentiation programs within tumor cells, changes in neural components of the tumor microenvironment, or a combination of both. While bulk methylation data do not allow these possibilities to be disentangled, the consistency across datasets argues against stochastic methylation drift.

#### 4.2.1 ZIC1 and ZIC4 as representative developmental hubs

ZIC1 and ZIC4 exemplify this group of hypermethylated developmental regulators. Both genes encode zinc-finger transcription factors with established roles in neural development (Aruga et al., 1994; Grinberg and Millen, 2005) and are frequently silenced by promoter CpG island hypermethylation in several cancer types (Gan et al., 2011; Ding et al., 2025; Wang et al., 2009; Chen et al., 2020; Paluszczak et al., 2017). In the present analysis, ZIC1 and ZIC4 were consistently hypermethylated at promoter-like CpGs across all cohorts. Although they are not established PDAC biomarkers, their recurrent epigenetic silencing places PDAC within a broader pattern of cancer-associated repression of neurodevelopmental transcription factors.

#### 4.2.2 Neuronal signaling and adhesion-related hubs

Other hypermethylated hubs, including GRIA4, GRIA2, CDH2, NCAM1, GDNF, NEUROD1, and TBR1, extend this theme to genes involved in synaptic signaling, cell–cell adhesion, and neurotrophic support (Crespo et al., 2022; Fielder et al., 2017; Li et al., 2021; Luo et al., 2022; Mulligan, 2019; Vivero et al., 2014; 2014; Zeng et al., 2025). Hypermethylation at promoter-proximal and enhancer-like regions linked to these genes suggests attenuation of regulatory elements that normally support neuronal communication and lineage specification. Given the importance of tumor–nerve crosstalk in PDAC, these methylation changes may be relevant to disease progression, although their functional consequences remain to be determined. Consistent with this concept, prior studies have shown that epigenetically regulated transcription factors and developmental regulators can influence tumor proliferation, migration, and immune interactions, providing a functional link between epigenetic state and oncogenic behavior (Marei, 2025; Topper et al., 2020). Emerging evidence in PDAC further suggests that regulatory programs may intersect with tumor–immune phenotypes; for example, SATB2-associated transcriptional networks have been linked to modulation of the tumor microenvironment and immune-related pathways (Jiang et al., 2025). While such mechanisms were not directly tested here, they support the potential biological relevance of the identified methylation patterns.

#### 4.2.3 Enhancer-associated hypermethylation of lineage regulators

Several key hubs, including OLIG2, NEUROD1, SLC32A1, and CDH2, were predominantly associated with enhancer-like hypermethylated CpGs. In neural tumors, these genes are regulated by active enhancer networks that maintain progenitor-like states (Desai et al., 2025; Li et al., 2021; Luo et al., 2022; Tsigelny et al., 2016). The presence of enhancer-associated hypermethylation at these loci in PDAC raises the possibility that distal regulatory elements linked to neural or developmental programs are selectively repressed. Whether this contributes to lineage restriction or subtype-specific transcriptional programs in PDAC requires direct experimental testing.

#### 4.2.4 Hypomethylated hubs are enriched for metabolic and stress-response genes

In contrast, hypomethylated hub genes were mainly associated with metabolic regulation, stress response, apoptosis signaling, and transcriptional control. Genes such as CPT1A, MCCC2, DUSP1, TNFRSF10A, PML, MAZ, and RUNX1 occupied central positions in the network and showed recurrent hypomethylation across datasets.

Among these, CPT1A is particularly notable, as its role in fatty-acid oxidation and PDAC progression has been supported by recent experimental and clinical studies (Liu et al., 2023; Yang et al., 2022; Yang et al., 2023). Hypomethylation at enhancer-like CpGs near CPT1A across all cohorts is consistent with epigenetic activation of metabolic pathways that promote tumor growth and metastasis. Other hypomethylated hubs, including DUSP1 and TNFRSF10A, may similarly reflect adaptation to cellular stress, although the regulatory mechanisms linking methylation to expression remain to be clarified (Niture et al., 2025; Xu, 2025).

Compared with hypermethylated CpGs, hypomethylated sites were distributed across a broader range of chromatin states, including enhancer-like and unmarked regions. This heterogeneity may partly explain their reduced consistency as predictive features in machine-learning models.

### 4.3 Limited overlap between hypomethylated CpGs and machine-learning features

Despite their biological relevance, none of the consensus hypomethylated CpGs overlapped with CpGs jointly prioritized by both machine-learning models. This observation is consistent with the regulatory-context analysis, which showed that hypomethylated CpGs are distributed across heterogeneous chromatin states, including enhancer-like and quiescent regions, rather than being concentrated at promoter-associated regulatory elements.

Such heterogeneity may limit the consistency with which hypomethylated loci contribute to tumor–normal classification across cohorts (Pharo et al., 2025). In contrast, hypermethylated CpGs are more frequently located in promoter-proximal or promoter-flanking regions, which are more directly linked to transcriptional regulation and tend to show greater stability across tumors. These features likely make hypermethylated CpGs more informative for predictive modeling, whereas hypomethylation may reflect subtype-specific, microenvironment-related, or context-dependent epigenetic changes (Ehrlich, 2019).

### 4.4 Differences in feature selection between machine-learning models

Only 52 CpGs were shared between features prioritized by SHAP-based logistic regression and those identified by the DeepPGD model. This limited overlap likely reflects differences in model architecture and feature attribution rather than disagreement regarding biologically relevant signals. Logistic regression emphasizes linear and additive contributions of individual CpGs, whereas deep learning models can capture nonlinear and distributed patterns across features (Levy et al., 2020; Yap et al., 2021). Importantly, CpGs prioritized by both approaches were enriched in promoter-like and enhancer-associated chromatin states, indicating that shared features tend to occupy regulatory contexts with direct roles in gene expression control. CpGs identified by only one model are often mapped to more diverse or context-dependent chromatin states, highlighting complementary but distinct aspects of the methylation landscape captured by each modeling strategy.

### 4.5 A compact 18-CpG panel integrating reproducibility and prediction

By intersecting SHAP-prioritized CpGs, DeepPGD-selected CpGs, and consensus hypermethylated CpGs, we derived a compact panel of 18 CpGs mapping to 25 genes. These CpGs consistently distinguish tumor from normal samples across all cohorts and represent features supported by statistical reproducibility, predictive relevance, and regulatory-context annotation.

Chromatin-state analysis showed that the majority of CpGs in this panel are located within promoter-like or enhancer-flanking regions, providing a regulatory annotation context for their consistent selection by machine-learning models. Although not all mapped genes correspond to major network hubs, several display moderate connectivity and may function as intermediates within larger regulatory networks (Hu et al., 2021; Ibrahim et al., 2023). Importantly, the objective of this intersection-based strategy was not to capture all potentially relevant CpGs, but to identify features that remain stable across heterogeneous cohorts and platforms.

While cohort-specific CpGs may reflect context-dependent or subtype-specific biology, such signals may be less reliable for cross-cohort prediction. Accordingly, although this approach identifies CpGs that are consistently observed across cohorts, it may preferentially select highly stable features and does not necessarily capture the most biologically impactful or cohort-specific methylation events.

To further examine this potential limitation at the cohort level, we compared consensus CpGs with cohort-specific differentially methylated CpGs (excluding shared loci) using pathway enrichment analysis. This analysis showed that both consensus and cohort-specific CpGs are associated with similar categories of biological processes, particularly neurodevelopmental and signaling-related pathways, although these associations are based on enrichment analyses and do not establish functional effects of methylation changes. However, cohort-specific CpGs displayed greater variability and reduced cross-cohort reproducibility, especially among hypomethylated loci.

These findings suggest that the intersection-based approach does not arbitrarily select statistically conserved features, but rather identifies a reproducible subset of CpGs within broader pathway-level patterns observed across datasets. Accordingly, the consensus CpGs identified in this study should be interpreted as a stability-focused core set suitable for cross-cohort biomarker development, whereas cohort-specific CpGs may warrant further investigation in stratified or subtype-focused analyses.

### 4.6 Interpretability of CpG contributions using SHAP

**FIGURE 11:**
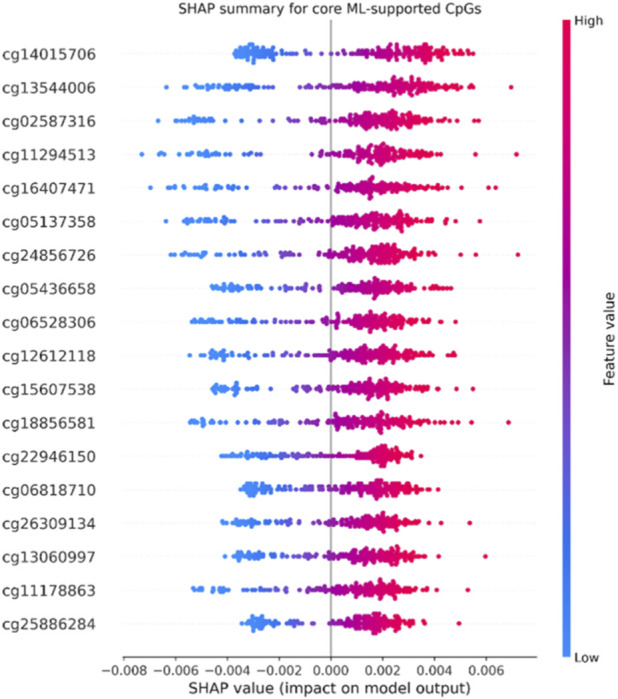
SHAP summary plot of core machine-learning–supported CpG sites in PDAC. Beeswarm plot showing SHAP values for the 18 core CpG sites jointly supported by SHAP-based logistic regression, DeepPGD-like modeling, and cross-cohort differential methylation analysis. Each point represents an individual sample, with the x-axis indicating the SHAP value (feature contribution to the PDAC versus normal prediction) and color denoting the corresponding CpG methylation level (blue: low; red: high). Positive SHAP values indicate increased contribution toward PDAC classification, whereas negative values indicate association with normal samples. The consistent directional effects across samples highlight the stability and interpretability of these CpGs as high-confidence predictive epigenetic features.

SHAP analysis enabled interpretation of the direction and relative magnitude of CpG contributions to model predictions. Most CpGs in the final panel showed positive contributions consistent with tumor-associated hypermethylation, while a smaller subset exhibited inverse effects (Figure 11). Integration with chromatin-state annotation indicates that these contributions predominantly arise from CpGs located in promoter-like or promoter-adjacent regions, supporting their functional relevance in transcriptional regulation.

CpGs showing inverse or nonlinear effects are often mapped to enhancer-like or non-promoter contexts, suggesting that their contributions may reflect context-dependent regulation or interactions with other genomic features rather than direct gene silencing. These results underscore the complexity of methylation-based classification and highlight the value of combining explainable modeling with regulatory annotation.

## 5 Limitations and future directions

This study has several limitations. Chromatin-state annotations were derived from reference pancreas tissue and may not fully capture PDAC-specific regulatory landscapes or tumor-induced chromatin remodeling. Bulk methylation data do not distinguish tumor-intrinsic epigenetic signals from stromal, immune, or neural contributions, which may particularly affect hypomethylated regions mapped to heterogeneous regulatory contexts.

However, it is important to note that network centrality analyses do not establish functional causality, and the absence of matched transcriptomic or chromatin accessibility data limits direct inference of whether observed methylation changes causally influence gene expression or downstream cellular phenotypes. In addition, independent orthogonal validation of selected CpG loci using targeted bisulfite sequencing or pyrosequencing was not performed in this study. Because the analysis relied on harmonized multi-cohort public datasets, residual cohort-specific effects and shared preprocessing structure cannot be fully excluded, and apparent robustness may therefore be somewhat inflated relative to fully independent validation settings. Future studies should integrate DNA methylation with RNA-seq, ATAC-seq, and histone-modification profiling in matched samples, apply locus-specific epigenome editing to evaluate the functional consequences of key CpGs, and validate selected loci using targeted methylation assays in independent PDAC cohorts, ideally including independently processed adjacent normal tissues. Accordingly, the identified CpGs should be interpreted as high-confidence candidate biomarkers prioritized for downstream validation rather than clinically validated markers.

## 6 Conclusion

Overall, these results indicate that PDAC is characterized by coordinated epigenetic repression of neurodevelopmental and lineage-associated regulatory programs, accompanied by more variable hypomethylation affecting metabolic, stress-response, and microenvironment-related pathways. Network-based analyses identified a set of topologically central hub genes enriched for neuronal and developmental functions, including regulators of neurogenesis, cell identity, and synaptic signaling. The recurrent hypermethylation of these hubs across independent cohorts suggests that disruption of neuronal-like regulatory programs represents a structured and conserved component of PDAC epigenetic remodeling, highlighting lineage plasticity and neuronal trans differentiation as important biological features for future investigation.

Moreover, integration of differential methylation analysis with machine-learning approaches identified a compact 18-CpG signature that consistently distinguished tumor from normal samples across four independent cohorts. This signature was supported by both linear and non-linear predictive models and showed preferential localization to promoter-like and enhancer-associated regulatory elements, providing a mechanistic basis for its cross-cohort reproducibility and robustness. Together, these findings indicate that PDAC-associated DNA methylation changes converge on stable regulatory features and position the 18-CpG set as a candidate predictive marker panel that requires independent orthogonal validation, such as targeted bisulfite sequencing or pyrosequencing in external PDAC cohorts, before clinical or biological robustness can be established.

## Data Availability

Publicly available datasets were analyzed in this study. This data can be found here: The datasets can be found on Gene Expression Omnibus site https://www.ncbi.nlm.nih.gov/geo/ and https://ega-archive.org/datasets/EGAD00010002386.
